# DEVELOPMENT OF DEMENTIA FRIENDS FOR INCARCERATED SETTINGS CURRICULUM

**DOI:** 10.1093/geroni/igac059.923

**Published:** 2022-12-20

**Authors:** Bonnie Burman, Elizabeth Kinzig

**Affiliations:** Ohio Council for Cognitive Health, New Albnay, Ohio, United States; Ohio Council for Cognitive Health, New Albany, Ohio, United States

## Abstract

This presentation describes the working relationships and steps that were taken to develop Dementia Friends for Incarcerated Settings. This included identifying population needs, approval processes, curriculum development specific to this population, learner coordination, implementation, measurement and reporting. Dementia Friends, a one-hour session to help everyone in a community understand five key messages about dementia, was used as the framework for an educational program targeting staff at correctional facilities. Dementia Friends is a standardized program that can be modified depending on the target audience or sector. Our sector-specific program was piloted in all 27 Ohio prisons to ensure that all staff are knowledgeable about ways to make a difference in the lives of those living with dementia.

